# Hypoinsulinemic hypoglycemia with hemihypertrophy in a Filipino infant: management in a resource-limited setting

**DOI:** 10.1210/jcemcr/luag231

**Published:** 2026-10-13

**Authors:** Elizabeth Grace M Perez, Sylvia C Estrada

**Affiliations:** Division of Pediatric Endocrinology, Department of Pediatrics, University of the Philippines—Philippine General Hospital, Manila 1000, Philippines; Division of Pediatric Endocrinology, Department of Pediatrics, University of the Philippines—Philippine General Hospital, Manila 1000, Philippines

**Keywords:** AKT2, hypoketotic hypoglycemia, fasting intolerance, overgrowth syndrome, infant

## Abstract

Persistent infantile hypoglycemia is a medical emergency associated with significant risk of neurological injury. Although most commonly attributed to congenital hyperinsulinism, disorders of impaired insulin signaling with hypoketotic hypoglycemia despite low or undetectable insulin occur in fewer than 1 in 1 000 000 infants worldwide, leading to misdiagnosis and ineffective therapy. We report a Filipino infant with recurrent fasting hypoglycemic seizures and hemihypertrophy. Biochemical evaluation showed absent ketones with low to mildly detectable insulin, suggesting dysregulated insulin signaling. In a resource-limited setting without access to diazoxide or advanced imaging, clinical and biochemical evaluation guided management. Genetic testing later confirmed a pathogenic *AKT2* variant (c.49G>A; p.Glu17Lys). Euglycemia was achieved through individualized nutritional strategies including uncooked cornstarch and nocturnal enteral feeding. This case highlights clues to *AKT2*-related hypoinsulinemic hypoglycemia with hemihypertrophy (HIHGHH), demonstrates that low insulin does not exclude insulin-mediated hypoglycemia, and underscores the importance of careful clinical evaluation alongside molecular diagnosis to guide management and prevent neurologic sequelae.

## Introduction

Persistent hypoglycemia in infancy is a metabolic emergency associated with a high risk of long-term neurodevelopmental morbidity, requiring prompt recognition and accurate etiologic classification [1, 2]. Neonatal hypoglycemia occurs in 1.3 per 1000 live births worldwide and reported in 1675 neonates among 5 998 507 cases in the Philippines as of January 2026 [2, 3]. Infants are particularly vulnerable due to limited glycogen stores, immature counter-regulatory mechanisms, and non-specific clinical presentation [1].

Although congenital hyperinsulinism is the most common cause, some infants present with hypoglycemia and suppressed ketogenesis despite low or undetectable serum insulin or C-peptide, suggesting impaired fasting adaptation due to dysregulated insulin signaling rather than inappropriate insulin secretion [1]. Insulin homeostasis is mediated through the phosphatidylinositol-3-kinase (PI3K)-AKT-mTOR signaling pathway; where *AKT2* is a key mediator [4-6]. Activating *AKT2* variants cause constitutive signaling, leading to insulin-independent glucose uptake, suppressed ketogenesis, and persistent hypoglycemia [7, 8]. Fewer than ten cases of hypoinsulinemic hypoglycemia with hemihypertrophy (HIHGHH) have been reported worldwide, with limited data especially in resource-limited settings [7-10].

We report a Filipino case, highlighting diagnostic and management challenges and the role of clinical reasoning and nutritional strategies in the absence of standard diagnostics.

## Case presentation

The patient was born term to non-consanguineous Filipino parents following an uncomplicated pregnancy and delivery. She was large for gestational age (LGA) with weight of 3.88 kg (>90th percentile), length of 51 cm (75th-90th percentile). Original neonatal glucose records could not be retrieved; however, available referral records, indicated routine glucose monitoring without documented hypoglycemia. Routine newborn screening was unremarkable and she was discharged on day 3 of life. She remained asymptomatic until 5 months old. Thereafter, episodes of pallor and blank staring developed after 4-5-hours of fasting.

Initial consultation led to admission for presumed aspiration pneumonia. During admission, she experienced her first generalized tonic-clonic seizure. Capillary blood glucose (CBG) was 30 mg/dL (SI: 1.7 mmol/L) (normal fasting range, 70-99 mg/dL [SI: 3.9-5.5 mmol/L]). Attempts to wean intravenous dextrose resulted in recurrent hypoglycemia, prompting referral to pediatric endocrinology. She was initiated on milk-cornstarch solution (1:1 dilution) every three hours and discharged without baseline critical sampling.

Following discharge, recurrent symptomatic hypoglycemia persisted with CBG values ranging from 28 to 49 mg/dL (SI: 1.5-2.7 mmol/L) characterized by decreased activity typically before the first morning feed or after 3-4 hours fasting, and transiently resolved with feeding. At six months old, episodes culminated in a second generalized tonic-clonic seizure at CBG 23 mg/dL (SI: 1.3 mmol/L), prompting admission to a children's hospital. Critical sampling (Table 1) revealed undetectable serum insulin at <1 μIU/mL (SI: <6.9 pmol/L) (reference range, 1-2 μIU/mL [SI: 6.9-13.9 pmol/L]), with otherwise normal metabolic parameters. Despite glucose-supplemented milk-cornstarch feeds every three hours, hypoglycemia recurred. The family subsequently sought second opinion at our institution; where she presented in active hypoglycemic seizure at CBG 36 mg/dL (SI: 2 mmol/L).

**Table 1:** Summary of critical samples

Critical Sample	(5/16/24) CBG 45 mg/dL (SI: 2.5 mmol/L)	(5/27/24) CBG 36 mg/dL (SI: 2.0 mmol/L)	Reference Range (conventional and SI units)
Insulin	**<1 μIU/mL** **(<6.9 pmol/L)**	**5.48** **μIU/mL** **(38.1 pmol/L)**	1-2 μIU/mL (6.9-13.9 pmol/L)
Cortisol	457 nmol/L (16.6 μg/dL)	**1225.25 nmol/L** **(44.4** **μg/dL)**	138-690 nmol/L (5-25 μg/dL)
Growth Hormone	NA	6.57 ng/mL (6.57 μg/L)	0-20 ng/mL (0-20 μg/L)
Urine Ketones	Negative	Negative	Negative
Ammonia	64.3 μg/dL, (37.79 μmol/L)	—	18.7-63 μg/dL (11-37 μmol/L)
Lactate	NA	0.91 mmol/L (8.2 mg/dL)	<1 mmol/L (<9.0 mg/dL)
C-peptide	NA	1.14 ng/mL (0.38 nmol/L)	0.8-3.1 ng/mL (0.3-1 nmol/L)
Arterial Blood Gas	NA	pH 7.36 pCO_2_ 34 mmHg (4.5 kPa) HCO_3_ 18.8 mmol/L	pH 7.35-7.45 pCO_2_ 35-45 mmHg (4.7-6.0 kPa) HCO_3_^−^ 22-26 mmol/L

Abnormal values are shown in bold font. Values in parenthesis are International System of Units (SI).

Abbreviations: HCO_3_^−^, bicarbonate; NA, not available; pCO_2_, partial pressure of carbon dioxide.

Pre-morbid, she tolerated formula feeds (1:1 dilution) every 2-4 hours and small amounts of pureed complementary food. Following recurrent hypoglycemia, regression in gross motor milestones, hypotonia, feeding intolerance, and loss of early vocalization were observed. Family and environmental history were unremarkable.

## Diagnostic assessment

On admission, she was irritable, hypotonic, with fair cry. She was tall-for-age (72 cm, z-score >+2), overweight (9.5 kg, weight for length (WFL) z-score >+2) with a head circumference of 44 cm (50-98th percentile). Dysmorphisms included bilateral supra-orbital fullness, infraorbital creases, flat nasal bridge, macroglossia, hepatosplenomegaly, and hemi-hypertrophy of the left lower extremity (1.5 cm longer than the right) (Fig. 1A).

**Figure 1:**
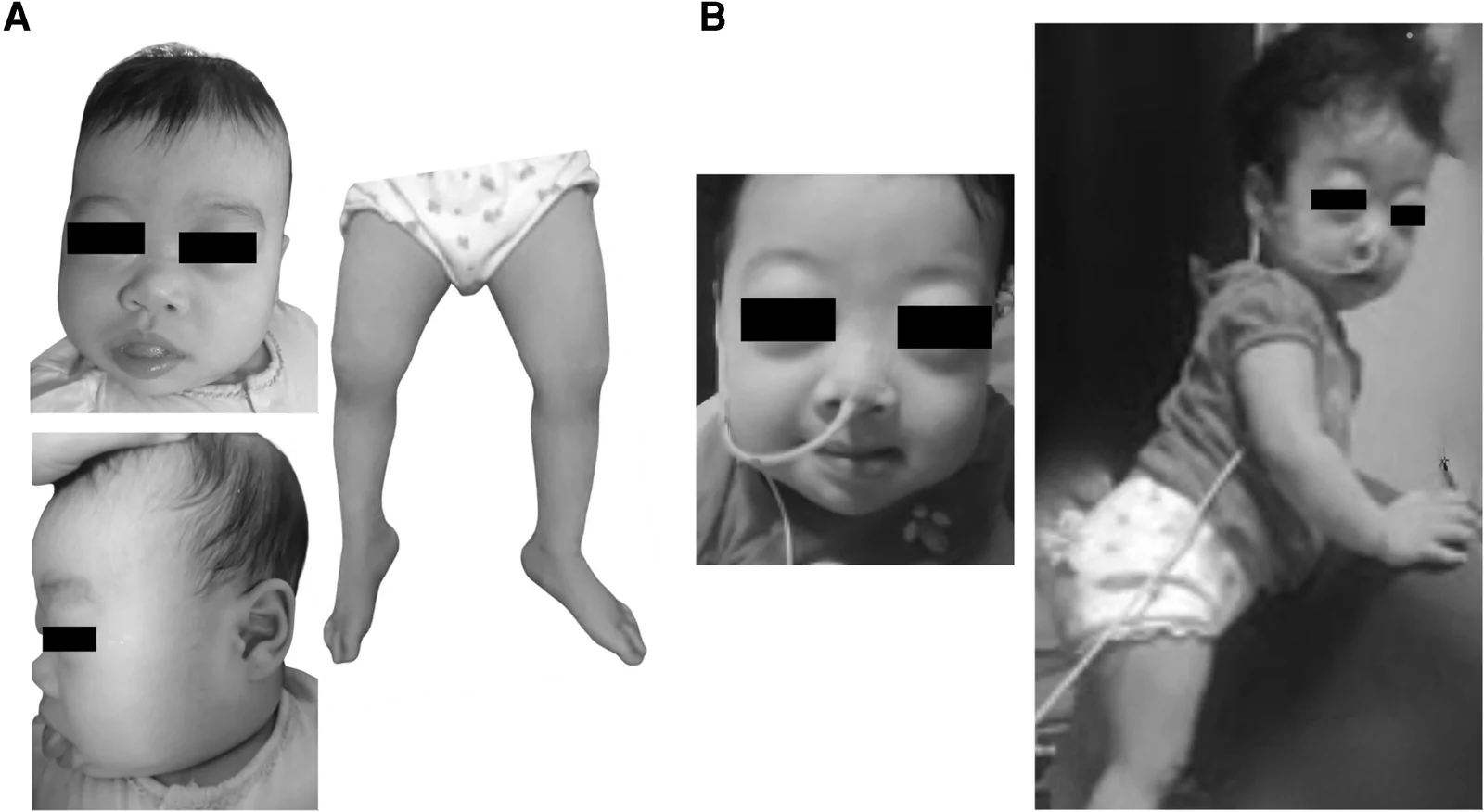
Clinical phenotype and follow-up (parental consent obtained): A) at 6 months: supra-orbital fullness, infraorbital creases, flat nasal bridge, macroglossia, and left lower extremity hemihypertrophy; B) at 16 months: improved muscle tone and developmental interaction.

Primary consideration included hyperinsulinemic hypoglycemia from Beckwith-Wiedemann syndrome or a monogenic etiology. Repeat critical sampling revealed mildly elevated serum insulin of 5.48 μIU/mL (SI: 38 pmol/L) and elevated cortisol of 1222.25 nmol/L (SI: 44.4 μg/dL) (reference range, 5-25 μg/dL [SI: 138-690 nmol/L]), without ketosis, and otherwise unremarkable metabolic parameters (Table 1). Plasma free fatty acids and β-hydroxybutyrate measurements were unavailable because of local reagent limitations. Diagnostic diazoxide trial was not performed, since it was also unavailable locally and biochemical features suggested insulin-independent hypoglycemia.

Fluorodopa positron emission tomography was unavailable, thus abdominal computed tomography with pancreatic protocol was done demonstrating non-enlarged pancreas without focal lesions, with incidental left hepatic lobe enlargement. Cardiac imaging revealed mild mitral regurgitation, prominent thymus, and incidental macroglossia. Given the persistent hypoglycemia with overgrowth, molecular testing was pursued confirming a pathogenic *AKT2* gene c.49G>A (p.Glu17Lys) variant verifying HIHGHH.

Targeted next-generation sequencing using a 169-gene hypoglycemia/overgrowth syndrome panel identified a heterozygous pathogenic *AKT2* variant (NM_001626.4: c.49G>A; p.Glu17Lys) consistent with autosomal dominant HIHGHH. This missense variant has been reported as de novo causing constitutive insulin-independent *AKT2* activation. A heterozygous MTOR variant of uncertain significance (c.4613G>A; pArg1538Gln) was identified but was not linked to the phenotype. *AKT2* variants have also been associated with susceptibility to type 2 diabetes mellitus, suggesting age-dependent metabolic consequences. Parental testing and mosaicism analysis were not performed due to financial constraints.

## Treatment

Management relied on frequent feeding and cautious reduction of glucose infusion rate (GIR). Persistent feeding intolerance and inability to tolerate a GIR below 3-4 mg/kg/min necessitated nasogastric tube (NGT) feeding at 2-3 hour intervals under gastroenterology guidance. Euglycemia (patient's CBG ranges: 68-149 mg/dL [SI:3.8-8.3 mmol/L]) was eventually achieved through 2-hourly uncooked cornstarch-milk feeds and continuous nocturnal enteral nutrition. Despite counseling, family declined gastrostomy placement.

## Outcome and follow-up

She was discharged on hospital day 62 with residual hypotonia. Multi-disciplinary and family conference emphasized strict feeding schedules, age-appropriate nutrition, and close monitoring of growth and neurodevelopment.

At 16 months, our patient showed accelerated weight (z-score >+2) and WFL (z>+2) with improved tone (Fig. 1B) without hypoglycemia though still requiring <2-hour milk intervals and nocturnal enteral nutrition. Ongoing metabolic and developmental follow-up underscores the chronic nature and need for multidisciplinary surveillance.

## Discussion

Euglycemia is maintained through a coordinated neuroendocrine response, with insulin suppression at plasma glucose (PG) <85 mg/dL (SI: 4.7 mmol/L), followed by glucagon and sympathetic activation at 65-70 mg/dL (SI: 3.6-3.9 mmol/L), and later contributions from cortisol and growth hormone to sustain glycogenolysis and gluconeogenesis [1, 2]. Impaired ketogenesis compromises these defenses and predisposes to severe and prolonged hypoglycemia. Critical samples obtained at PG <50 mg/dL (SI: 2.8 mmol/L) remain essential in differentiating endocrine, metabolic, and syndromic etiologies of persistent hypoglycemia [1, 2, 11].

In our patient, low to mildly detectable insulin with absent ketosis and preserved counterregulatory hormones (Table 1) suggested dysregulated insulin signaling [11]. Insulin receptor activation normally triggers the PI3K-AKT-mTOR pathway, with *AKT2* regulating glucose uptake and suppression of gluconeogenesis [4-6, 12]. The p.Glu17Lys gain-of-function variant enables constitutive, insulin-independent activation by binding to the more abundant phosphatidylinositol-4,5-bisphosphate [7, 13]. This results in persistent suppression of gluconeogenesis and ketogenesis despite low insulin, explaining hypoketotic hypoglycemia unresponsive to conventional therapies for hyperinsulinism [11, 13]. Sustained glucose uptake and anabolic signaling further parallel mechanisms in overgrowth syndromes [9, 14, 15].

Evaluation of persistent infantile hypoglycemia should follow established algorithms correlating critical sample interpretation and clinical phenotype [1, 11]. In hypoketotic hypoglycemia with low or inappropriately detectable insulin, disorders of insulin signaling, including *AKT2*-related disease, should be considered after exclusion of fatty acid oxidation defects and glycogen storage disorders. Clinically, recurrent fasting hypoglycemia with overgrowth should prompt consideration of insulin signaling disorders.

In our setting, limited access to diazoxide, advanced imaging, and prompt genetic testing necessitated adaptation of standard algorithms with reliance on clinical and biochemical pattern recognition to guide targeted testing and management, enabling timely intervention despite delayed genetic confirmation.

Table 2 summarizes previously reported cases of HIHGHH associated with the *AKT2* p.Glu17Lys gain-of-function variant [7-10]. First described in 2004 with molecular confirmation in 2011, both heterozygous and mosaic variants demonstrate a consistent phenotype with characteristic dysmorphism and requirement for continuous enteral feeding [7-10, 16]. Excess downstream signaling, compounded by caloric intake, predisposes to obesity, femoro-gluteal lipodystrophy, hepatic steatosis, and tall stature [17]. Hemihypertrophy is biologically plausible because *AKT2* is a key mediator of the PI3K-AKT-mTOR pathway regulating cellular growth and proliferation. Constitutive pathway activation likely promotes asymmetric tissue overgrowth, analogous to PI3K-AKT pathway overgrowth syndromes. Whether mosaic and heterozygous *AKT2* variants contribute to variable expression remains uncertain [4-6, 13-15].

**Table 2:** Clinical comparison of reported *AKT2* p.Glu17Lys cases [7-10]

		Hussain et al, 2011 [7]			Arya et al, 2014 [8]	Garg et al, 2015 [9]	Molina et al, 2022 [10]	Our patient
		Proband A	Proband B	Proband C				
Zygosity		Heterozygous	Heterozygous	Mosaic	Mosaic	Heterozygous	Mosaic	Heterozygous
Gestational age, Birth weight (KG), Percentile (*P*)		Term 4.5 kg, *P* > 99	Term, 4.51 kg, *P* > 99	Term, 3.4 kg, P70	41 weeks, 3.9 kg, P78	Term NA	Term, 3.8 kg, P75	40 weeks, 3.88, *P* > 90
SEX		Male	Male	Female	Female	Female	Male	Female
Age at presentation (months)		12	15	5	5	6	6	5
Presenting symptoms		Seizures	Hypotonia, pallor, lethargy	Seizures	Reduced consciousness	Lethargy, Flat forehead	Seizure, macrocephaly,	Pallor, blank stare
Dysmorphisms		Left-sided hemihypertrophy		Facial asymmetry	Macrocephaly, left hemihypertrophy	hypertrichosis, prominent eyes, hypertelorism, flat nasal bridge, macroglossia mild hepatomegaly	hypertelorism, proptosis, flat nasal bridge	proptosis, infraorbital crease, flat nasal bridge, macroglossia, left-sided hemihypertrophy
Fasting intolerance (≥ 3 hours)?		Yes	Yes	Yes	Yes	Yes	Yes	Yes
Critical sample Treatment	Glucose	39.6 mg/dL (2.2 mmol/L)	28.8 mg/dL (1.6 mmol/L)	28.8 mg/dL (1.6 mmol/L)	43 mg/dL (2.4 mmol/L)	27-34 mg/dL (1.5-1.9 mmol/L)	38 mg/dL (2.1 mmol/L)	36 mg/dL (2.0 mmol/L)
	Insulin	< 1 μIU/mL	< 0.4 μIU/mL	< 0.3 μIU/mL	< 2 μIU/mL	< 0.2 μIU/mL	<0.2 μIU/mL	5.48 μIU/mL
	C-peptide	NA	NA	NA	NA	< 0.1 ng/mL	<0.033 ng/mL	1.14 ng/mL
	Free Fatty Acids	8.6 mmol/L	9.6 mmol/L	4.6 mmol/L	NA	NA	NA	NA
	β-Hydroxy butyrate	0.4 mg/dL (0.04 mmol/L)	1.04 mg/dL (0.10 mmol/L)	<0.52 mg/dL (<0.05 mmol/L)	≤ 0.52 mg/dL (<0.05 mmol/L)	NA	1.45 mg/dL (0.14 mmol/L)	NA
	IVF (GIR)	4 mg/kg/min	4 mg/kg/min	NA	NA	Bolus (as needed)	3-4 mg/kg/min	3-4 mg/kg/min
	Interval (hours) between feeding	2	2	3	Regular (bedtime cornstarch)	2 (cornstarch every 4 hours)	2-3	2
	Continuous enteral feeding	Yes	Yes	No	No	No	Yes	Yes
Out come	Oldest reported	17 years	17 years	3.5 years	4.3 years	1 year 6 months	8 months	1 year 4 months
	Anthropometrics (SD based on WHO growth chart)	BMI 28.5 kg/m^2^, (+0.7 SD) Body fat 37%	BMI 22.3 kg/m^2^ (+0.7 SD) Body fat 37%	(18 months): Weight P91, Length P75	Weight P91 Length P98	BMI +0.8 SD Height +2 SD	WFL +3.9 SD Length +1.7SD Head circ +3.8	WFL +1 SD Weight +2SD Length +2SD
	# of hours fasting tolerated	12	< 3	< 3	6	< 3		< 3
	Other findings	Elevated serum leptin, Normal trigylcerides, low VLDL, low HDL	Elevated serum leptin. Normal trigylcerides, low VLDL, low HDL	Elevated serum Leptin (28.1 µg/L)		Gross motor delay Acanthosis nigricans		Feeding intolerance, verbal and gross motor delay

Values in parenthesis are International System of Units (SI).

Abbreviations: BMI, body mass index; GIR, glucose infusion rate; HDL, high-density lipoprotein; IVF, intravenous fluid; NA, not available; P, percentile; SD, standard deviation score; VLDL, very-low-density lipoprotein; WFL, weight-for-length; WHO, World Health Organization.

Although our patient's critical sample demonstrated mildly detectable insulin and measurable C-peptide, these remained inappropriately low for the degree of hypoglycemia and contrasted with classic congenital hyperinsulinism. In *AKT2* gain-of-function disorder, constitutive activation of downstream insulin signaling produces insulin-like metabolic effects despite low insulin, underscoring the importance of interpreting critical samples within the broader biochemical and clinical context, including insulin and C-peptide concentrations, suppressed ketogenesis, and characteristic phenotype [7-11, 17].

Currently, no established targeted therapy exists for AKT2 related HIHGHH (Table 2) [7-10]. Patients are unresponsive to diazoxide or octreotide. Management focuses on preventing fasting through frequent carbohydrate intake, avoiding >3 hours fasting, requiring continuous overnight feeds, with uncooked cornstarch as slow-release glucose adjunct. When enteral feeding is contraindicated, GIR 2-4 mg/kg/min is beneficial [18]. Waxy maize heat-modified starch for glycogen storage disease showed no superiority [10]. Sirolimus (0.5 mg/m^2^), an mTOR inhibitor, modestly prolongs fasting (4-5 hours) but needs further investigation [17].

Caregivers should be educated to recognize early neuroglycopenic symptoms. Age- and culturally-appropriate nutrition, especially during the first 1000 days of life, are essential and were implemented for our patient in accordance with local guidelines [19].

Most reported cases are diagnosed during later infancy, likely reflecting delayed recognition rather than delayed disease onset. Frequent feeding in early infancy may initially mask fasting intolerance, while the rarity of *AKT2-*related disease, its relatively recent recognition, and limited access to genetic testing may delay diagnosis. Limited evidence also suggests that mosaic and heterozygous *AKT2* variants contribute to phenotypic variability, including differences in disease severity and age at presentation [1, 7-10, 13, 17].

Long-term outcomes remain incompletely defined due to HIHGHH's rarity (Table 2) [7-10]. One 17-year-old demonstrated improved overnight fasting tolerance, whereas others with similar genotypes continue intensive nutritional support despite normal hepatic and low plasma triglyceride, highlighting phenotypic variability [7, 17]. Somatic *AKT2* pathogenic variant has been reported in malignancy, but not with the p.Glu17Lys variant [4]. Neurodevelopmental outcome appears closely related to hypoglycemia severity, recurrence, and diagnostic delay.

## Learning points

- *AKT2* gain-of-function expands infantile hypoketotic hypoglycemia.
- In resource-limited settings, pragmatic recognition and nutritional management is central.
- Early clinical recognition and molecular diagnosis guide targeted management and avoids ineffective treatment.
- Lifelong growth and neurodevelopmental surveillance is required.

## Data Availability

Some or all datasets generated and/or analyzed during the study are not publicly available but are available from the corresponding author on reasonable request. The pathogenic *AKT2* variant (c.49G>A; p.Glu17Lys) is a previously reported variant archived in ClinVar (Variation ID: 29804; VCV000029804.4). No novel sequencing data was generated in this study.
